# Peer specialists and mental health nurses who work with patients who are suicidal: A comparative interview study

**DOI:** 10.1016/j.ijnsa.2024.100285

**Published:** 2024-12-23

**Authors:** Diana D. Van Bergen, Tove Henseler

**Affiliations:** Department of Pedagogical and Educational Sciences, University of Groningen, Groningen, the Netherlands

**Keywords:** Suicidal behavior, Peer specialists, Mental health nurses, Peer support, Suicide prevention

## Abstract

**Background:**

In the field suicide prevention, knowledge about the involvement and approaches of peer specialists is scarce, prompting an examination of their potential unique contributions compared to what mental health nurses offer.

**Objectives:**

We compared perspectives of peer specialists, mental health nurses, and patients with suicidal thoughts) on: 1) ‘causes’ of suicidality, 2) essential skills, insights, and interactions in working with patients who feel suicidal; and 3) beneficial approaches for reducing suicidality.

**Design:**

Qualitative interviews with three types of informants were analysed thematically using the Constant Comparative Method. The samples, all from the Netherlands, consisted of 19 peer specialists with a history of suicidality, 18 mental health care nurses, and seven patients with suicidality who had been in contact with both peer specialists and mental health nurses.

**Results:**

All three groups viewed suicidality as a prolonged process driven by problematic situations and thoughts, primarily to escape life rather than die. All groups found the following important: suicide literacy (i.e., knowing what it means to be suicidal and what is optimal suicide care), empathy, and understanding. Patients, however, felt peer specialists showed greater unconditional empathy than nurses, likely because nurses focused on risk assessment and safety. Patients also found peer specialists more convincing in promoting recovery from suicidality than nurses, with their lived experiences serving as powerful examples.

**Conclusion:**

Both mental health care nurses and peer specialists articulated suicide literacy and understood the relevance of empathy and genuine listening in caring for patients who feel suicidal. Nevertheless, in practice, nurses are less often experienced as empathic by patients and do not always abide by shared decision making (due to prioritising risk- and safety assessment). Through their own previous suicidal crises, peer specialists are unique in their ability to break down hierarchical barriers with authentic empathetic support.

**What is already known about the topic**

• Although there is evidence of the effectiveness of peer specialists in mental health care, knowledge on their role in the field of suicide prevention is scarce.
• It is unclear what the potential unique contributions of peer specialist are compared to mental health nurses in suicide care and whether this resonates with patients’ views.

Alt-text: Unlabelled box

**What this study adds**

• Both mental health care nurses and peer specialists articulate suicide literacy, but in practice nurses are less often perceived as understanding by patients who feel suicidal.
• Peer specialists are unique in their ability to break down hierarchical barriers with authentic empathetic support.
• Patients who feel suicidal should be offered the opportunity to have contact with both with mental health nurses and peer specialists.

Alt-text: Unlabelled box

## 1 Introduction and background

Worldwide, >700,000 people die by suicide each year, while a far greater number entertain suicidal thoughts (Nock et al., 2008; WHO, 2023). A sizeable proportion of suicides in many western countries – an estimated 50–60 % – concern adults who have received treatment for mental health problems (Stene-Larsen and Reneflot, 2019). It is therefore crucial that patients using mental health services are offered good quality care to provide an adequate response to their suicidality (i.e., suicidal attempts and thoughts) and thus to prevent suicides (Kapur et al., 2022). Mental health nurses are of particular relevance since they are among the staff that spend significant amount of time with patients in a suicidal crises and its aftermath, to promote wellbeing and safety (Clua-García et al., 2021).

Many mental health care nurses recognize that their capacity to empathise, listen, and observe and to be in the proximity of the patient and available are critical to help patients who are suicidal (Clua‐García et al., 2024). Nevertheless, many mental health nurses state that their training does not always make them feel equipped to work with patients who are suicidal, because suicidality is either not discussed during their training or, if it is, only theoretically (Scupham and Goss, 2020). This does little to demolish the taboos surrounding suicidality, even after nurses have qualified. Some researchers have shown that nurses find patients in suicidal distress challenging and emotionally intense (MacGarry et al., 2022; Vandewalle et al., 2019a). Researchers have shown that, where mental health care workers have gained more years of work experience, without receiving appropriate training in suicide prevention, this is not accompanied by greater perceived self-efficacy in suicide prevention (Dundas et al., 2022). In addition, during their contact with a patient who is suicidal, mental health nurses are required to compile a treatment plan and suicide risk assessment, which can make face-to-face discussion with a person who feels suicidal more difficult in the time-limited setting of the treatment session (Huisman and van Bergen, 2019; Vandewalle et al., 2019a).

## 2 Peer specialist services in mental health care and suicide prevention

Over the last decade, there has been an increase in many western countries in the deployment of ‘peer specialists’. i.e., people who upon (certified) training turn their previous experiences with mental health or psychiatric problems (here: particularly with suicidality) and their recovery into the ability to assist in the recovery, healing and self-management of other care consumers who struggle with mental health challenges, including suicidality (Bellamy et al., 2017; Huisman and van Bergen, 2019; Klee et al., 2019). There has been a visible increase in the use of peer specialists in suicide prevention (Hawgood et al. et al., 2023), although there are differences in their employment across countries and between different mental health care institutions (Hawgood et al., 2023; Schlichthorst et al., 2020). Peer specialists have in most cases received training to learn how to help in the recovery and healing (hereafter ‘recovery’ is used for brevity reasons) of people with suicidal thoughts or mental health problems and how they can bring their own lived experiences to bear in a professional manner (Klim et al., 2022; Ng and Barlas, 2023).

To date, little scientific research has been carried out on the working methods and effectiveness of peer specialists in suicide prevention (Hawgood et al., 2023). However, peer support (for a variety of psychiatric problems or illnesses but not for suicidality specifically) within mental health care was found effective for outcome measures related to clinical recovery and personal recovery in a recent meta-analytical study (Smith et al., 2023). In their meta-analysis, Smith et al. (2023) demonstrated that peer specialists working with patients alongside or within regular treatment programmes, were effective in enhancing personal recovery, particularly referring to elements such as hope (or decreasing hopelessness) and giving meaning to life (Smit et al., 2023) (Pfeiffer et al., 2019; Salzer et al., 2016).

Qualitative researchers have identified the perceived values of peer specialists in suicide prevention (Ng and Barlas, 2023; Schmutte et al., 2023). In a review, Schlichthorst et al. (2020) examined what specific content and mechanisms underlie the work of peer specialists in suicide prevention. An important factor is the reciprocal understanding between peer specialists and mental health patients regarding their mental anguish (i.e., suffering over adversities in life, negative thoughts and emotions) and wish to die, which may courage openness and the ability to discuss shared mutual experiences of despair (Bergmans et al., 2009; Chi et al., 2014; Sun and Long, 2013). Mutual understanding may enable bonding, between the peer specialist, a form of connectedness that may buffer against suicidal behaviour, as suggested by the Interpersonal Theory of Suicide (Joiner, 2005). Moreover, the peer specialist's recovery can serve as an example for the patient because the peer specialist is living proof that things can get better in the future, even if the patient is unable to see this at the time (Kirkegaard, 2022). These findings concerning recovery also align with the Social Learning theory proposed by Bandura (1977; see also Klee et al., 2019); i.e., the notion that people draw inspiration for their behaviour from what they observe around them among people meaningful to them.

## 3 Peer specialists’ work compared to that of mental health nurses

Peer specialists might be in a better position than mental health nurses (without lived experience) to work with patients in suicidal distress, given their good starting position in terms of acknowledging and discussing suicidality and breaking down stigma (Schlichthorst et al., 2020). However, it is important not to be too quick to draw conclusions here, given the aforesaid fairly limited corpus of available research. Moreover, there are a number of potential pitfalls in the deployment of peer specialists in a suicide prevention role with mental health patients (Hawgood et al., 2023; Huisman and van Bergen, 2019). The contact between a peer specialist and a suicidal patient may be (too) onerous for the peer specialist if they themselves are not sufficiently recovered (Schlichthorst et al., 2020). Moreover, looking at the professional qualities of mental health nurses with no lived experience of suicidality, it might be expected that, with training and work experience, together with their motivation for working as a mental health care professional, they too should be able to make effective contact with patients who are suicidal (Clua‐García et al., 2024).

Given that there is ambivalence surrounding the unique added value of peer specialists, it is important to gather more empirical knowledge about the substantive work that peer specialists do in relation to suicide prevention, and how this may differ from mental health nurses. The aim of this study was therefore to contribute to a nascent body of empirical research into optimising suicide prevention care by identifying how peer specialists, mental health nurses, and mental health care patients view the ‘causes’ to suicidality and, in particular, what constitutes good care for patients who feel suicidal (e.g., skills, knowledge, interaction, approaches for reducing suicidality). Our research questions are: 1) What meanings are attached to feeling suicidal? 2) Which skills, insights, and interactions are crucial for working with patients who feel suicidal?, and 3) What are beneficial approaches for reducing and recovery from suicidality within the context of mental health care? By answering these three questions by systematically addressing perspectives of peer specialists (with lived experience with suicidality) with those of mental health care nurses and patients, we hoped to provide more clarity about how unique the type of treatment and support offered by peer specialists was compared to mental health nurses. As a result, we hoped to have contributed to the optimisation of care for patients in suicidal distress among mental health nurses.

## 4 Method

### 4.1 Context of the study

We focused on the Netherlands where, (as in other Western countries) within mental health care, there has been growing attention for using peer specialists in suicide prevention, even though this is not yet happening in a systematic or uniform way (Huisman and van Bergen, 2019). Daily work tasks of peer specialists resemble several aspects of the work that mental health nurses also often do; for example social activities, and chats with patients, offering proximity when they feel suicidal, co-leading recovery-oriented group sessions. However, some employers restrict peer specialists to providing only social activities, for example.

Regarding the treatment (and diagnosis) of suicidal behaviour, mental health care professionals are required offer optimal suicide care, which means working in accordance with the multidisciplinary guideline for suicidal behaviour (Van Hemert et al., 2012). The guideline (Van Hemert et al., 2012) argues that deploying a range of techniques can reduce the risk of suicidal behaviour; they include making good contact with the patient, daring to question the patient about their suicidality and its origin, and adopting a supportive, empathic and transparent approach whilst acknowledging the patient's suffering.

### 4.2 Sample and recruitment of participants

For the recruitment of peer specialists, one institution was selected at random from the 30 largest mental health care institutions in each of the 12 provinces of the Netherlands; that institution was then approached and asked if any peer specialists working with patients who feel suicidal would be willing to take part in the study. This resulted in the inclusion of 19 peer specialists in the study: 14 women and five men, ranging in age from 26 to 66 years. The participants were evenly distributed across the 12 Dutch provinces. In addition, a minority (five participants) came from the researchers’ networks. The peer specialists’ training varied from a 2-year associate peer specialist degree (university training), to a certificate of attendance for a short course. Those degrees and courses were designed specifically for people with lived experience who want to be employed as peer specialist in mental health care. All peer specialists had themselves been suicidal in the past, and were focused on both mental health and suicide prevention in their work. Of the 19 peer specialists, 14 worked in mental health care institutions, and five had done so in the recent past. Most of their work took places in clinical settings, but sometimes it involved home visits.

A similar method was used for the recruitment of mental health nurses; from a random selection of 35 large mental health care institutions operating across the 12 Dutch provinces, two to three organisation were approached in each province with the question whether mental health nurses would be willing to take part in an interview. A precondition for participation was that they were in regular contact with patients who were suicidal. A total of 18 mental health nurses participated. They comprised 11 women and seven men ranging in age from 28 to 63 years. They had worked in the mental health care service for an average of 19 years in the roles of group leader (*n* = 1), nursing specialist (*n* = 1), nurse (*n* = 3), graduate practitioner nurse (*n* = 3), or social psychiatric nurse (*n* = 10). None of the nurses reported that they had themselves experience a suicidal period (but were not asked either; see limitations).

The 7 patients were recruited through four national platforms and self-help organisations focusing on suicide prevention, as well as via mental health care institutions. A condition for inclusion was that the patients (had) suffered from suicidality (ideation or attempts) and were receiving treatment for this in the mental health care system and had been in direct contact with a peer specialist. Four of the participating patients were women, and three men; three were aged between 20 and 35 years, while four were between 36 and 64 years old. Participants received a voucher of 20 euros for participating.

### 4.3 Instrument and interview procedure

We interviewed the three groups of informants using a semi-structured topic list, in which the topics were largely the same. All three groups were, for example, asked about their views on suicidality, their take on the most appropriate care intervention in relation to suicidality and what they believed the (added) value would be of deploying peer specialists in cases of suicidality. Nurses and peer specialists were also asked about their experiences in working with patients who feel suicidal, while patients were asked about their own experience of suicidality, and the help they received from a peer specialist. There were three interviewers, all female, in their twenties, thirties and fifties respectively, with backgrounds in psychology, pedagogy, and psychiatric nursing, with a variety of familiarity with mental health care institutions. The interviews were held face-to-face in a separate room in the mental health care facility and lasted between 45 min and 1.5 h. Quotations in Dutch from the interviews were translated to English by a certified translator. The research procedures received ethical clearance from the Medical Ethics Committee at the University of Groningen.

### 4.4 Analysis

All interviews were recorded and transcribed verbatim in Dutch and subsequently subjected to thematic analysis using the ATLAS.ti program (version 21) (Braun and Clarke, 2014). The coding of the transcripts was carried out by two researchers (also the authors). Initially, each coder coded a number of interviews independently and then compared their results. The first coder then continued, and their work was checked by the second coder. A combination of deductive and inductive coding was used. Examples of deductive coding (where codes are based on the interview instrument) include, ‘What are helpful forms of care intervention for patients who feel suicidal?” An example of inductive coding (where codes are based on topics not explicitly part of the interview instrument) is, ‘capability and braveness to discuss suicide and suicidal thoughts’. The list of principal codes was then expanded and often subdivided into several subcodes following consultation and discussion between the two researchers. As far as possible, the principal codes were systematically constructed in the same way for all three informant groups, with the exception of a few topics to which only one or two respondent groups were able to give an answer. We first coded each interview by informant type. The interviews were then compared across informant types using the previously constructed codes (Boeije, 2002). This process continued until a saturation point was reached, and no new principal codes or subcodes could be added to the substantive text fragments. In the analysis phase, the data were initially analysed per group and subsequently systematically compared across the three types of informants. A form of member checks was established by presenting preliminary results of the interview findings at a small symposium, followed by small adjustment to the results.

## 5 Results

### 5.1 Views on the ‘Causes’, onset, and existence of suicidality

#### 5.1.1 Peer specialists

The meanings attached by peer specialists to suicidality among their patients included anxiety about life, a lack of perspective and meaning, and a desire to change the lives that people were currently leading. Several peer specialists reported that all manner of processes preceded suicidal behaviour; situations could become so complex that people lost their ability to see the big picture or no longer felt connected to others. In many cases, peer specialists argued that suicidality was therefore a coping strategy: patients’ lives had become more than they could bear and they saw suicidality as a way of escaping things they no longer wanted to feel or go through. According to peer specialists, suicidality could also stem from a wide variety of psychiatric illnesses, and this diversity determined the course of the suicidal process and its treatment:

‘I've come across suicidality from lots of clinical pictures. And they're completely different. If you don't know that and treat them as being all the same or try to deal with them in the same way, you'll get it completely wrong.’

The different illnesses that peer specialists associated with suicidality included depression, autistic spectrum disorders, personality disorders, and psychotic disorders. One peer specialist also noted that many people, after having thought frequently about suicide or after making a first attempt, have become familiar with this ‘escape route from life’, and this can lower the threshold to making a (repeat) suicide attempt. The same peer specialist also stated that patients who have gone through a suicide attempt regularly say that they are unwilling to commit totally to whether or not they wish to continue living; the peer specialist reported that she can empathise with this very strongly.

#### 5.1.2 Mental health nurses

Like peer specialists, nurses observed that suicidal behaviour can stem from an accumulation of factors, and they too, saw a relationship between suicidality and various psychiatric illnesses. Again similarly to peer specialists, nurses noted that suicidality could be a coping strategy and could mask a desire for something different in and from life but without any idea of how to achieve this. Nurses also frequently saw loneliness and isolation among patients who felt suicidal, which was in line with the observed lack of connectedness referred to by peer specialists. Care professionals noted that a stressful life event and (associated) psychosocial problems could be related to the onset of suicidality, again in line with the observation by peer specialists regarding the difficult situations in which their patients found themselves. Yet there are also important differences. Unlike peer specialists, mental health nurses did not emphasise anxiety about life as a key factor in suicidality but instead placed the emphasis on despair and helplessness. Moreover, unlike peer specialists, mental health nurses stressed that the majority of patients who were suicidal did not have a genuine death wish but above all desired peace, whereas peer specialists, whilst also seeing this, less readily rejected the idea of a death wish on the part of their patients, possibly because they knew the depth of mental pain from their own first-hand experience.

#### 5.1.3 Patients

The views of patients reflected elements cited by both peer specialists and mental health nurses. Like peer specialists, patients felt that anxiety played a central role in suicidality, but unlike peer specialists, they linked this anxiety explicitly to their thoughts, rather than to life in general. They cite anxiety about their own thoughts and about themselves, and describe how suicidality can come to completely dominate their thoughts, which they then found themselves constantly having to battle. In addition, patients described the despair mentioned by nurses as characteristic of suicidality. One patient described suicidality as, *‘one big, black hole, and you're no longer capable of seeing beyond it’*. In line with what mental health nurses suggested, several patients indicated a desire for peace. They also endorsed the ambivalent feelings about death highlighted by mental health nurses in particular as being a part of suicidality; not so much wishing to be dead but mainly not feeling able to live with the problems they were wrestling with at a given moment and wanting to be free of them: '*You just want to escape. I just wanted to sleep forever. It wasn't so much that I wanted to be dead, more that I just wanted to get away from it. I just wanted peace and quiet.'*

Like peer specialists, patients also said that the route to suicidality became easier, more tangible and more real the longer it played in their thoughts. Several patients found that suicidal thoughts seemed to come almost unbidden, and that it was difficult to ignore those thoughts after an earlier suicide attempt: '*At some point you've taken the decision that you can leave this place if you want;(…) Once the gate's open, I don't think you can ever close it again completely'.* Several patients (in line with nurses), also reported not just life events or situations (in line with peer specialists), as the reasons for a suicide attempt, but also ‘*no longer feeling (anything)*’, and ‘*falling out of contact*’. The latter statement corresponded with the lack of connectedness mentioned by peer specialists. In contrast to both peer specialists and nurses, not a single patient made a link between their suicidality and a psychiatric illness. This could be related to the fact that patients were also the only group to emphasise the young age from which they began wrestling with suicidal feelings, perceiving trauma in childhood or adolescence in particular as an important reason behind their suicidal thoughts.

### 5.2 Skills and insights needed for working with patients with suicidal thoughts

#### 5.2.1 Peer specialists

According to peer specialists, a key skill when working with patients who were suicidal was the ability to make a genuine connection. Peer specialists saw added value here in their ability to empathise with the patient from the basis of their own experiences: '*Being there. Sharing the same experience of pain. That's what being a peer specialist is about.'* Skills which peer specialists believed were at least as important were being able to give patients a sense of being accepted as human beings and offering them acknowledgement and recognition of their pain. As another peer specialist explained: '*(…) the emotional connection. Giving warmth. The feeling that the person matters'. Even if they are on the point of killing themselves.* This peer specialist argued that peer specialists possess unique skills precisely because of their experience of recovery from suicidality, both regarding their ability to make connections and being a source of hope. Peer specialists also said that their own past experience of suicidality meant they were able to talk to patients about suicidality without inhibitions, while patients told them that this was often not the case with mental health nurses who had not experienced recovery from suicidality.

#### 5.2.2 Mental health nurses

Like peer specialists, nurses stressed the importance of acknowledging the suicidality and the pain of patients who felt suicidal and of establishing a good connection with them. They also described empathy and person-centredness as an important quality. Where peer specialists drew on a lived experience with suicidality to achieve that connection, nurses relied on approaching the patient openly and calmly, their ability to listen well and ‘being there’, thus creating space for a dialogue about suicidality: '*Being there, being available; [suicidal] patients are naturally at a very low point and need something to hold on to, something they are not able to find in their immediate setting, or at least not enough.'* Another technique used by mental health nurses was to try and establish a connection was being a genuinely good listener, being genuinely present, focusing attention on the patient and genuinely wanting to hear what they are saying, checking that the patient and care professional understood each other and helping the patient reach a greater insight.

Regarding safety, mental health nurses mentioned a different skill from peer specialists; namely, reflecting and being responsive to their gut feeling or a sense that ‘something doesn't feel right’. One care professional cited the example of a patient who ‘put on a brave face’ following a suicide attempt:

'(..) there was something; I can't really say exactly what it was that gave me the feeling of ‘I just don't trust this’. And luckily the psychiatrist had exactly the same feeling (…). So we admitted the patient (…) [Later] he said he was glad we had decided he needed to be admitted because otherwise he would have died by suicide that day.'

#### 5.2.3 Patients

Interestingly, patients largely considered the same skills to be important as peer specialists and nurses; they too mentioned the importance of acknowledging and recognising their suicidality and their pain, offering closeness by being there for them, talking openly and above all empathically about suicide, and the importance of creating a calm atmosphere during a suicidal period. Several patients also stressed two further skills which resonated particularly with what was stated by peer specialists. The first was the peer specialists’ openness about their own suicidal experiences, which patients found to be very appropriate and comforting because it countered their sense of shame. Patients found that this openness by peer specialists can reduce the taboo and sense of discomfiture: '*I found it very difficult to trust people and be open, partly through shame, but she was also very open about what she had gone through.'*

The second skill mentioned by patients was the importance of being treated like a human being. This fits in with peer specialists’ belief in the importance of seeing/perceiving patients as people. Patients also think this should go hand in hand with giving less priority to protocols or their medical files. Finally, there is also one item that was mentioned particularly by patients; namely the importance of one aspect of suicide literacy (knowledge of what it means to be suicidal), so that carers are able to ask the patient sensitively but directly about suicidality.

### 5.3 Interaction with mental health care patients around suicidality

#### 5.3.1 Peer specialists

Several peer specialists stressed the importance of being able to talk to patients openly and directly about suicidality and the associated thoughts and emotions. Acknowledging the other person's suicidal thoughts and the pain that lies behind them; accepting the patient as a person and accepting all of their feelings; listening to them without immediately proffering advice, panicking, taking action or assessing risks against a protocol, were all of great importance in peer specialists’ view. They also liked to create space for the patient in their interaction with them, and to explore ways in which the situation could be eased somewhat. They did this by homing in on what caused the patient to have these thoughts, but also by showing their acknowledgement of the patient's situation by referring to their own past suicidality, as well as their recovery from it. According to peer specialists, it was important that patients are seen and heard in what they felt, thought and said about their suffering and their desire to end their lives.

#### 5.3.2 Mental health nurses

Like peer specialists, nurses stressed the importance of talking openly with their patients about suicidality. Openness can come as a relief; as one nurse puts it:

'(…) those are good moments for me; when I start a conversation and just talk about things and ask them about things like keeping safe; then I mention the word suicidality, or thinking of suicide, and those are moments when people often… you often see that they break and start talking for the first time.'

Like peer specialists, mental health nurses stressed the importance of empathy towards the patient's experience of suicidality, and that this could create a space to talk about suicidality:

'So I start by saying, ‘God, how awful for you that you feel like that; I can absolutely, totally understand that you feel powerless’. Just by saying that, you can often create an opening for people that makes them think, ‘oh, it's OK for me to talk about it.'

Like peer specialists, mental health nurses stressed the importance of asking directly about the patient's thoughts and plans and of gaining an insight into why the patient was feeling suicidal. They added that it was important to consider what each individual patient needed to feel safe enough to talk about their situation.

#### 5.3.3 Patients

Like peer specialists and nurses, patients also stressed the importance of being open about suicidality, though there was a discrepancy here: the vast majority of patients said they were not able to talk to mental health nurses (enough) about their deathwish during their treatment. The following quote from a patient illustrates their experience that there were few opportunities for openness about suicidality:

'I never had the feeling that there's genuine scope within the mental health care system to have a real conversation about death. (…) But you do have to be able to talk about it because it's a very big part of me. Every day.'

Like peer specialists, several patients felt that nurses relied heavily on protocols and that this could sometimes get in the way of genuine attention and space to discuss the patient's suicidal thoughts and plans. One patient reported feeling that they were not taken seriously when they shared their suicide plans and that following a subsequent suicide attempt, they were referred to a different institution because their suicidality was ‘too intense’. Several patients said that this protocol-based approach and their perception of being judged, not taken seriously, or with little opportunity for openness caused them to keep their suicidal thoughts to themselves. Most patients who came into contact with a peer specialist said they were better able to talk about suicidality and to feel understood. Patients also reported that mental health nurses tended to focus on admission or coercion; one patient referred to the focus on action, a desire to ‘save’ the patient, rather than allowing the (suicidal) feelings to ‘just be there’ and contrasted the closeness they felt with the peer specialist:

'In the FACT (Flexible Assertive Community Treatment) programme, I always had the feeling that I had to perform. (…) I knew what I was supposed to say. (…) I always felt that remoteness, that hostility. I didn't feel any of that with [the peer specialist]. They weren't trying to save me; they just wanted to stand by my side.'

Patients experienced a greater sense of calm with peer specialists, more space, and a feeling that they can ask more questions. They felt comfortable with this kind of interaction. According to one patient, being able to reflect on suicidal thoughts with a peer specialist helped them understand what their suicidality actually represented: '*The more I thought about it, the more I began to see that I still couldn't live with the trauma; it was more that than genuinely wanting to be dead.'*

### 5.4 What are helpful forms of care intervention for mental health care patients who felt suicidal ?

#### 5.4.1 Peer specialists

According to peer specialists, being on an equal footing with the patient and once having been suicidal themselves and recovered, was helpful because it gave patients a sense of hope for recovery. One peer specialist recounted how he used his experience of recovering from suicidality to pique patients’ curiosity about the possibilities for recovery: '*(…) that they (the patient) start asking me about how it was for me. And I say that there was a time when I too no longer wanted to go on, but that I'm glad I'm still here. ‘So what happened?’*

In a similar vein, another peer specialist explicitly sought to show the patient that recovery is also possible for them: Giving a feeling of hope. You can be an example, like ‘I've been in the same situation and come out the other side. You can do that too.

Peer specialists also felt it was helpful for patients if suicidal feelings could be discussed very openly and if they were listened to properly. They believed this openness could make the suicidal thoughts less overwhelming and less intense. Peer specialists also emphasised the importance of working with the patient if divulging their suicide plans led to protocol-based action, including restricting their liberty. As one peer specialist put it: '*Engage in dialogue with patients and tell them you have a sense that they're finding it difficult to make a decision at that moment, and suggest that you look at it together rather than concluding that they don't have capacity and taking control over them.'* Without this collaboration with the patients, their loneliness increased, according to the peer specialists.

On a practical level, many peer specialists also mentioned a specific method that they regard as helpful for recovery from suicidality; namely the *Wellness Recovery Action Plan,* this is a self-help recovery method that involves drawing up a plan for teaching appropriate individual skills, as well as tips on how to involve others from your social network. Peer experts valued this method for patients who were suicidal because it encouraged hope and promoted empowerment, both of which were needed to counter the feelings of helplessness and powerlessness that often accompanied suicidal thoughts.

#### 5.4.2 Mental health nurses

Interestingly, much of what mental health nurses regarded as helpful for a patient during a suicidal period corresponded with what peer specialists described as helpful, such as the fact that being open about suicide could paradoxically help patients to create more space for living or at least help to reduce the stress or ease the burden. Also like peer specialists, nurses described acknowledging the patient's suicidality and the pain that underlies it, and talking about the underlying ‘causes’ from a position of closeness, as helpful. They also stressed the need for openness about suicidal thoughts and feelings and of being a good listener, without immediately presenting possible solutions or taking measures. They also said it could be supportive to let patients know that they were not the only ones wrestling with suicidal thoughts. Like peer specialists, nurses stressed the importance of ensuring that the patient remained in control; however, they interpreted this differently from peer specialists. Rather than working together with the patient to look at restrictions of liberty if the patient had suicidal thoughts, they talked to them about learning to ask for help at an early stage and asking the patient themselves to set targets for their treatment. One care professional described it like this: '*If someone is suicidal, they'll have to learn how to deal with that, how to say ‘I don't feel good; I need to tell someone; I can make other choices; I can ask for help’.*

#### 5.4.3 Patients

In line with peer specialists and mental health nurses, patients also found it helpful when suicidality was acknowledged and referred to explicitly, and when they were able to talk about it openly to someone who was genuinely listening and reacted calmly. Also in common with peer specialists and to a lesser extent nurses, patients stressed the importance of retaining control over the care programme they received. However, by no means all patients thought that nurses acted in accordance with this. Whereas nurses indicated that control is agreed as far as possible in consultation and dialogue, the experience of many patients is that control stayed in the hands of the nurses. Patients did not have this feeling when working with peer specialists. In the words of one patient:

'She [peer specialist] allowed lots of space. She made it clear to me (…)‘you're in charge. You say what you want or don't want, and I'll work with you.’ My experience with [mainstream care provision] was often that I was told ‘you have to do this and that, you have to take this and that medication. We think you're not doing very well just now, so you're now going to be admitted.’

Like mental health nurses, patients referred to the importance of identifying what lay behind the suicidality and of feeling at ease with and having confidence in the contact with the care professional as being helpful for their suicidality. Similar to the other two groups, patients stressed the importance of mutual closeness, with patients who had contact with peer specialists stating that they felt they were treated more as equals. The shared experience created a sense of understanding each other and meant there is less need to explain things. Patients found this helpful and comfortable. Several patients said they found it helpful when caregivers were open about their own mentally stressful experiences but that it was almost exclusively peer specialists who displayed this openness. Moreover, peer specialists’ stories often offered the prospect that improvement was possible. As one patient put it: '*The peer specialist was open about her own suicidal thoughts and how she dealt with them. That gave me a bit of hope, as if there was a bit of light shining in the darkness.'* Like peer specialists, patients saw a Wellness Recovery Action Plan as helpful in making a switch towards recovery and picking up their lives again, including building a network.

Patients also mentioned a number of unique aspects that were helpful for recovery but were not identified by the other two groups. First, they wanted to see a greater focus in their treatment on those areas where they were still doing well – on their ‘healthy side’ – rather than being regarded only as a desperate or mentally ill person. Second, they found that greater flexibility in the length of sessions and better accessibility of formal carers helpful for their suicidality. Finally, patients were the only group who said they also needed carers who employ humour, including – or especially – at moments when they were having suicidal thoughts.

## 6 Discussion

The interest in peer specialists and an increased focus on recovery within mental health care has also led to (renewed) attention for what is required for genuinely good care (e.g., skills, knowledge, interaction, approaches for reducing suicidality) employed when working with patients who feel suicidal. First, we found a high degree of correspondence in the meaning that was attached to suicidality by peer specialists, nurses and patients. All three groups stressed that suicidality is a lengthy process, in which a concatenation of problematic situations and suicidal cognitions plunged people into a downward spiral, culminating in them wanting to end their lives. This perspective also aligns closely with the scientific literature which refers to ‘the suicidal process’, which generally unfolds gradually and in phases (Joiner, 2005). There is also consensus among all three informant groups that suicidality mainly signified not so much that a person wants to die, but more that they want to escape their present life with its desperate traumas and horrific thoughts. This too resonates with key findings from present-day suicidology (e.g., Kim et al., 2018). It was, however, notable that the peer specialists and patients in this study placed more emphasis than nurses on the chronic nature of suicidality and the fact that previous suicidal behaviour lowered the threshold to repeat a suicide attempt. This latter finding corresponds with the Van Heeringen et al. (2019) study on the course of repeated suicidal behaviour, as well as with Joiners (2005) theory of suicide, which posits that a previous suicide attempt can be viewed as enhancing a person's capability of suicide. Another difference in our study was that peer specialists mainly described the anxiety about life that they recognised in their patients as an underlying factor in their suicidality, whereas mental health nurses referred mainly to the desperation and sense of hopelessness. This difference could perhaps be explained by differences in the stage at which these two groups came into contact with patients; generally speaking, nurses are involved in cases of acute suicidality, when the patient's desperation is very manifest, whereas peer specialists often only come into the picture for a dialogue with these patients in non-acute situations.

Secondly, we investigated the skills and insights that were regarded as essential when working with suicidal mental health care patients. Once again, we found a good deal of agreement between the three informant groups; all three groups reported that adopting an empathic, understanding attitude towards suicidality, whilst at the same time reassuring the patient that it was acceptable for them to have those suicidal feelings, were central to working with people who were (acutely) suicidal. This aligns with the model developed by Podlogar et al. (2020), which uses the dimension ‘attitudes’ to indicate the potential tension between an open to accepting attitude towards suicidality by nurses and the ‘life-oriented’ approach used to try and influence the ambivalence felt by patients about life and death. The empathic approach frequently referred to by our respondents also aligns with the study by Vandewalle et al. (2020), in which nurses felt it was particularly important to regard the contact with patients as a relational process. This empathic approach is also in accordance with recommendations in the Guidelines on Suicidal Behaviour in the Netherlands (Van Hemert, 2012), which all caregivers in the Netherlands are expected to observe. Yet despite this, patients in our study reported that they found that peer experts offered greater empathy in their discussions about their suicidality than mental health nurses. It is likely that this stems from the priority which, according to Vandewalle et al., 2019a, Vandewalle et al., 2019b, some mental health nurses give to assessing and documenting the suicide risk of their patients and ensuring their safety. Patients in this study confirmed that they observed this tendency among mental health nurses.

Thirdly, we investigated how the three groups of participants assessed efforts to reduce suicidality. Peer specialists and patients highlighted a somewhat different approach here from mental health nurses. Their shared experiences of suicidality gave patients and peer specialists a sense that the peer specialists stood more *alongside* the patient and eschewed any form of hierarchy, suggesting more emphasis on shared or supported decision-making in suicide care. As a result, patients sometimes felt more heard and better understood and in some cases, also found it easier to overcome their feelings of shame, stigma and taboo in their contact with a peer specialist. It should, however, be noted here (see also Vandewalle et al., 2020) that suicidality is a topic that ultimately also requires nurses to have a willingness to act if a patient suddenly formed a genuine plan to die by suicide, which can cause hierarchy to find its way into the relationship. Hence, differences between nurses and peer specialists, including a tendency for substituted decision making, are also related to nurses’ responsibilities for the course and outcomes of the patient's treatment. Next, the patients in our study also found peer specialists to be more persuasive than mental health nurses in conveying the message that recovery from suicidal thoughts was definitely possible. Seeing a tangible example within a mental health care institution of someone who has plumbed the very depths of despair and yet still managed to find a meaningful role in life is a powerful signal for suicide prevention. This role model function of peer specialists is also described in a study by Kirkegaard (2022) and Klim et al. (2022).

## 7 Strengths and limitations of the study, and suggestions for further research

It proved to be difficult to find and recruit patients in mental health care who had been in direct contact with a peer specialist with lived experience with suicidality. The number of patients participants (*n* = 7) in the study, was therefore smaller than of peer specialists and nurses, yet just satisfactory according to the Guest et al. (2006) classic paper on saturation in interview studies. This reflects the fact that the use of peer specialists in suicide prevention is still in its infancy in mental health care in the Netherlands.

The nurses in this study described themselves with a more positive attitude towards patients displaying suicidal behaviour than was found in some other studies (Awenat et al., 2017, Vandewalle et al., 2019a, Vandewalle et al., 2019b). It is possible that mental health nurses who regard suicidality as a difficult topic surrounded by taboos did not volunteer to take part in our study; another possible explanation is that the competence of nurses has grown in recent years due to the increased attention being given to suicidality.

None of the care professionals interviewed for this study indicated that they themselves had ever felt suicidal. We therefore suspect that if they have had such an experience, they do not bring this to bear in their work. This is, unfortunately, a common practice (Victor et al., 2022). It is however a shortcoming in our interview instrument that we did not systematically raise this topic in our interviews; that is something that could be explicitly enquired after and discussed in future research.

In general, it would be interesting to conduct future research using a co-production methodology in a collaborative partnership with peer specialists, throughout the research process, so scientific progress can benefit more coherently from their voices and experiences.

## 8 Conclusions and implications for practice

In contrast to Awenat et al. (2017) and Vandewalle et al. (2019a), for example, we found many indications of nurses articulating empathic attitudes to patients with suicidal thoughts, emphasising the importance of understanding and listening to patients’ needs. Nevertheless, patients did not agree that nurses demonstrated a high level of competence in interacting with patients who felt suicidal; they described nurses as being primarily concentrated on safety and risk assessment. Hence, tensions may exist between nurses’ suicide literacy (i.e., what they know about optimal suicide care) versus what their institution demands of them on the work unit.

Peer specialists offer a unique form of care in that they are able to break through the hierarchy normally observed between care professionals and patients, which makes patients feel they are being invited to talk freely and openly about their suicidality. Peer specialists and patients confirm that they are both familiar with the struggle with suicidality from personal experience; this helps them to establish a good connection at both an emotional and relational level, potentially creating a space from which hope, and eventually, recovery can ensue (Kirkegaard, 2022; Schlichthorst et al., 2020). Hence, patients should be offered the opportunity to receive support from both a peer specialist, as well as from a mental health nurse. In addition, particularly those patients who perceive too much hierarchy in their contacts with mental health nurses, can reap benefits from having contact with a peer specialist.

## CRediT authorship contribution statement

**Diana D. Van Bergen:** Writing – review & editing, Writing – original draft, Supervision, Software, Resources, Project administration, Methodology, Investigation, Funding acquisition, Formal analysis, Data curation, Conceptualization. **Tove Henseler:** Writing – review & editing, Investigation, Formal analysis.

## Declaration of competing interest

None.
